# 109. Evaluating Predictive Value of Surgical Resected Proximal Bone Margins in Diabetic Foot Osteomyelitis with Clinical Outcomes at One Year

**DOI:** 10.1093/ofid/ofab466.109

**Published:** 2021-12-04

**Authors:** Bruce Weng, Yasmin Oskooilar, Bishoy Zakhary, Chiao An Chiu, Nikki Mulligan, Patrick Wu, Made Sutjita

**Affiliations:** 1 Riverside University Health System Medical Center, Redlands, CA; 2 Unversity of California, Riverside, Moreno Valley, California; 3 Riverside University Health System Medical Center - Comparative Effectiveness & Clinical Outcomes Research Center, Moreno Valley, California

## Abstract

**Background:**

Diabetic foot osteomyelitis (DFO) remains a significant comorbidity in diabetes and often requires both surgical and medical interventions. Surgical bone resection with proximal margins is performed for treatment at our institution to guide antimicrobial therapy. Optimal antibiotic duration often remains unclear, along with clinical outcomes with negative margins. We evaluate if negative bone margins predict outcomes of DFO at one year in our county hospital.

**Methods:**

A retrospectively cohort study assessed adult patients undergoing DFO amputations between 9/2016 to 9/2019. Patient data collected included demographics, smoking history, hemoglobin A1c (HbA1c), basic labs, microbiology, antibiotic duration, bone margin pathology. Physician review of records determined if intervention was successful. Primary outcome was met if no further amputation at the same site was required in the following 12 months.

**Results:**

Of 92 patients, 57 had negative margins and 35 had positive margins for pathology confirmed osteomyelitis. Smoking history was significant in positive margins (35.1% vs 57.1%; p=0.038). Patients with negative margins had a successful outcome at 12 months compared to positive margins (86% vs 66%; p=0.003), but no significant differences in outcome at 6 months. Antibiotic days was reduced in negative margin individuals (mean 18 vs 30 days; p=0.001). Negative margins also demonstrated significant lower rates of readmission at 12 months (p=0.015). S*taphylococcus aureus* was notable in positive vs negative margins (57.1% vs 29.8%; p=0.017). MSSA was significantly noted in positive margins (45.7% vs 14%; p=0.001). MRSA was similar regardless of margin results (15.8% vs 11.4%; p=0.399). Initial ESR, CRP and HbA1c were similar between groups.

**Conclusion:**

Our study noted that negative proximal bone margins resulted in more successful outcomes at 12 months and less days of antimicrobial therapy. Patients with negative margins had lower rates of readmission at 12 months for surgical site complications. Negative proximal bone margins results can guide antibiotic therapy and improve outcomes of resections. Presence of *S. aureus* was significant in positive margins and likely warrant consideration for further aggressive intervention.

Clinical Characteristics of Patients with Diabetic Foot Osteomyelitis

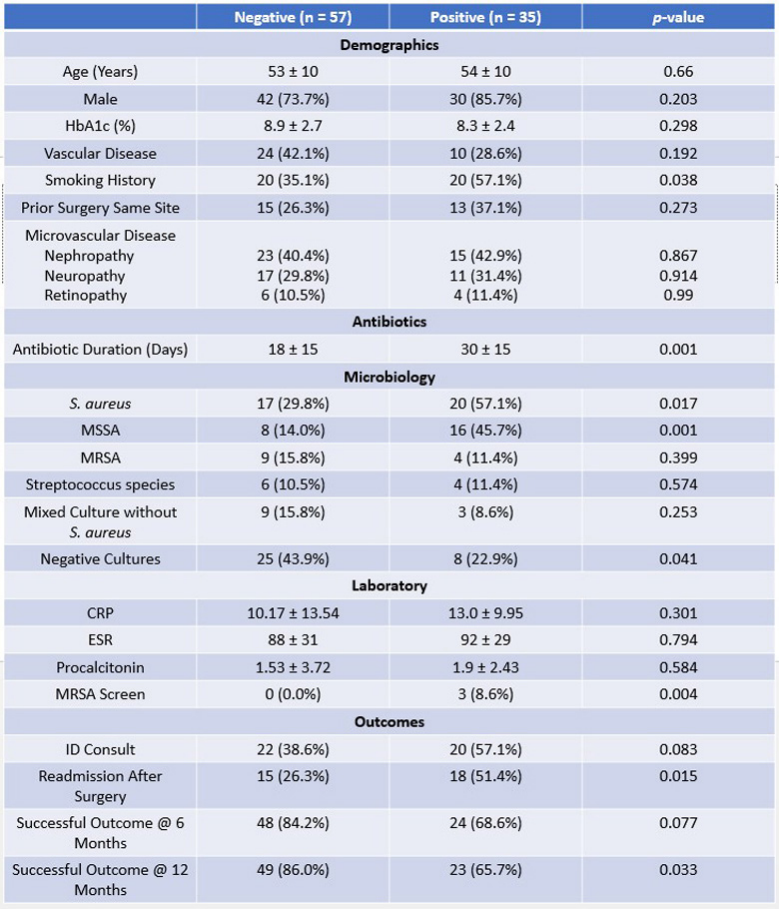

Clinical demographics, antibiotic usage, microbiology and results of patients presenting for diabetic foot osteomyelitis needing surgical amputation intervention. Abbreviations: HbA1c - Hemoglobin A1c; MSSA - methicillin-susceptible Staphylococcus aureus; MRSA - methicillin-resistant Staphylococcus aureus; CRP -C-reactive protein; ESR - erythrocyte sedimentation rate

**Disclosures:**

**All Authors**: No reported disclosures

